# Expectations and Concerns about the Use of Telemedicine for Autism Spectrum Disorder: A Cross-Sectional Survey of Parents and Healthcare Professionals

**DOI:** 10.3390/jcm11123294

**Published:** 2022-06-08

**Authors:** Alessandra Gabellone, Lucia Marzulli, Emilia Matera, Maria Giuseppina Petruzzelli, Anna Margari, Orazio Valerio Giannico, Lucia Margari

**Affiliations:** 1 Department of Basic Medical Sciences, Neurosciences and Sense Organs, University of Bari “Aldo Moro”, 70121 Bari, Italy; alessandra.gabellone@uniba.it (A.G.); lucia.marzulli@uniba.it (L.M.); maria.petruzzelli@uniba.it (M.G.P.); 2 Department of Biomedical Sciences and Human Oncology, University of Bari “Aldo Moro”, 70121 Bari, Italy; emilia.matera@uniba.it; 3 Interdisciplinary Department of Medicine, University of Bari “Aldo Moro”, 70121 Bari, Italy; margarianna2@gmail.com; 4 Department of Prevention, Local Health Authority of Taranto, 74121 Taranto, Italy; oraziovaleriogiannico@gmail.com

**Keywords:** telemedicine, telehealth, autism spectrum disorder, expectation, concern, barrier, benefit, healthcare professional, parent

## Abstract

Telemedicine has recently been used for diagnosis and interventions inpatients with autism spectrum disorder (ASD), traditionally performed in-person, but little attention has been paid to user expectations prior to its use. The aim of this study is to compare the expectations and concerns of 50 healthcare professionals and 45 parents of children with ASD regarding the use of telemedicine for diagnostic or treatment purposes. Parents have higher expectations for the use of telemedicine as an alternative (*p* = 0.0223) and supplement (*p* = 0.0061) to in-person diagnosis of ASD, as well as a supplement to traditional intervention (*p* ≤ 0.0001). In addition, while they also have greater hope for improvement in family routines (*p* = 0.0034) and parenting skills in child management (*p* = 0.0147), they express greater concern about the need for active parental involvement/supervision during telemedicine services (*p* = 0.015) and changes in the behaviour of the child with ASD during telemedicine services (*p* = 0.049). On the other hand, healthcare professionals are more concerned about barriers such as lack of devices (*p* = 0.000), unfamiliarity with the technology (*p* = 0.000), poor quality of internet connection (*p* = 0.006), and severity of ASD (*p* = 0.000). To achieve promising healthcare for ASD patients, the telemedicine service should try to meet the needs and preferences of both healthcare professionals and parents, as well as identify and, if possible, reduce perceived barriers.

## 1. Introduction

Autism spectrum disorder (ASD) is a neurodevelopmental disorder characterized by difficulties in social communication and interaction, as well as repetitive and stereotyped patterns of behaviour [1]. Once considered a rare condition, the prevalence of ASD has increased at an alarming rate according to recent epidemiological studies. According to data published by the Center for Disease Control and Prevention (CDC) in 2021, indeed, 1 in 44 children aged 8 years was diagnosed with ASD [2]. Advancement in diagnostic procedures, broadening of the diagnostic criteria, recommendations for universal screening for ASD, and increased public awareness of the disorder may partially justify the epidemiologic outbreak, suggesting that a true increase in the prevalence of ASD is conceivable [3,4]. Anyway, the increasing prevalence over time has led to an increased demand for diagnostic services for ASD [5].

ASD has no single known cause. Genetic, biological, and environmental factors interact with epigenetic mechanisms in the development of the disorder [4,6,7,8]. Several genes are associated with the pathogenesis of ASD, most of which are involved in synaptic dysfunction—environmental factors associated with the disorder include environmental pollutants, advanced parental age, chronic maternal infections and diseases, and medication use during pregnancy. Moreover, according to the literature, neuroinflammation and immune dysfunctions could play an etiologic role in ASD. A large body of research aimed at searching for correlations between ASD and impaired immune function [9,10,11] and found that familial autoimmunity, maternal autoantibodies, and inflammation during pregnancy may increase the risk for ASD. Consistently, higher titers of autoantibodies and rates of immune dysfunctions or immune-mediated comorbidities have been found in ASD individuals than in the healthy population, and processes of neuroinflammation have been demonstrated both ex vivo and in post-mortem brain samples [12], with an inflammatory response associated with oxidative stress mechanisms responsible for creating a microenvironment that can activate microglial cells in the brain.

To highlight the heterogeneity of symptoms, severity, and comorbidities associated with this disorder, the umbrella term “spectrum” was adopted [13]. Indeed, on one side of the spectrum there are children who lack any form of social reciprocity and verbal and nonverbal communication and whose behaviour is characterized by severe repetitive and stereotyped movements; on the other side, there are less impaired children with fluent but idiosyncratic verbal language. Between these two opposite extremes, there is a continuum of clinical presentations with very different characteristics. In individuals with ASD, there is also excessive adherence to routine with the child resisting change, even when minimal, with great variability from one individual to another, as well as sensory atypia resulting in children developing unusual responses to sensory stimuli (e.g., auditory, visual, tactile, gustatory, and olfactory), and various strategies to reduce physiological arousal are currently under investigation [14].

ASD diagnosis is essentially based on the patient’s developmental history and clinical observation of the child’s social communication and behaviour [15], since there are no biochemical tests or instrumental examinations that can identify an ASD diagnosis. However, some standardized assessment tools can be used to support ASD diagnosis, including the Autism Diagnostic Observation Schedule (ADOS) [16] and the Autism Diagnostic Interview—Revised (ADI) [17]. Other important components of the ASD diagnostic process include the assessment of cognitive, language, behavioural, and adaptive (daily living skills) functioning as well as screening for co-occurring medical and behavioural conditions, since ASD patients often have one or more comorbidities [18]. 

Although ASD is a life-long disorder, patients can benefit from early, ongoing, need-based, and multidisciplinary care, traditionally provided face-to-face [4,8]. More recently, social restrictions imposed during the coronavirus disease 2019 (COVID-19) pandemic, including isolation and lockdown, have led to a temporary suspension of diagnosis, treatment, and socio-educational care for ASD patients to minimize the risk of viral transmission [19]. In place of face-to-face care, healthcare professionals sought new telematic strategies to provide virtual care to their patients (also referred to as telemedicine, telehealth, or e-medicine), and several papers with practical advice have been published in the last few years [20,21,22,23,24,25]. The term “telemedicine” was coined by Thomas Bird in the 1970s and literally means “distant healing” [26]. Although there is currently no single definition for this new modality of healthcare delivery [27], it refers to the practice of caring for patients remotely when healthcare professionals (physicians, speech therapists, psychologists, etc.) and patients are unable to meet in-person for some reason (e.g., the COVID-19 emergency). As defined by the World Health Organization (WHO), telemedicine is “the delivery of health care services, where distance is a critical factor, by all health care professionals using information and communication technologies for the exchange of valid information for diagnosis, treatment, and prevention of disease and injuries, research and evaluation, and for the continuing education of health care providers, all in the interests of advancing the health of individuals and their communities” [28]. Many healthcare fields are currently using telemedicine, including psychiatry, cardiology, radiology, dermatology, physical medicine, ophthalmology, and pediatrics [29]. Telemedicine has been used in child and adolescent psychiatry since before the COVID-19 pandemic to provide diagnoses and interventions for neuropsychiatric disorders such as ASD. Preliminary studies were conducted to enable families in rural and remote areas to access health services, as they would otherwise have to travel long distances to centres with appropriate expertise [30,31,32,33]. The results of these studies highlighted the potential benefits of telematic support for ASD patients and encouraged healthcare professionals to use telemedicine in the recent outbreak when face-to-face meetings have become largely unfeasible. There are two main types of telemedicine, distinguished by the different timing of information transfer: synchronous, or real-time, telemedicine (which requires the simultaneous presence of the individuals involved); and asynchronous, or store-and-forward, telemedicine (which provides for the exchange of pre-recorded data between the individuals involved) [34]. Synchronous telemedicine allows healthcare professionals to communicate in real-time with ASD patients and their families and perform a virtual assessment or intervention, as in the case of videoconferencing. On the contrary, asynchronous, or store-and-forward, telemedicine allows healthcare professionals and the ASD patient’s family to communicate with each other by sharing multimedia materials. For example, after caregivers film videos of the child’s worrisome behaviour or fill out questionnaires, they send them to healthcare professionals or upload them to a platform or app [35,36]. 

However, research on the use of telemedicine to diagnose ASD is limited. Preliminary studies have used the Real-Time method to evaluate the accuracy of virtual assessment through traditional tools or adaptations of the same for remote diagnosis purposes. The results of these studies suggest that telemedicine is useful and valuable in the diagnosis of ASD, by comparing the virtual assessment using traditional tools with the previously performed in-person one or with the additional blinded one [37,38]. In addition, the COVID pandemic led to the implementation of clinical trials on the use of new tools. Among them, TELE-ASD-PEDS has been specifically developed for the remote caregiver-mediated observation of autism-related behaviours in young children under 36 months of age in order to support diagnostic decision-making (yes/no/maybe ASD) by an expert clinician who also administers a comprehensive developmental history and symptom-focused interview (clinicaltrials.gov, NCT03847337) [39]. At the same time, another clinical trial has been carried out to compare the effectiveness of the TELE-ASD-PEDS tool with another telehealth screening tool for children aged between 24 and 36 months, called TELE-STAT, although data are not yet available (https://clinicaltrials.gov/ct2/show/NCT03847337 accessed on 24 May 2022). Other preliminary studies aimed to demonstrate the accuracy of the store-and-forward method in diagnosing ASD. Following clinicians’ instructions, parents answered structured interviews or recorded short videos showing the child in four typical scenarios—(1) mealtimes, (2) playing with others, (3) playing alone, and (4) in specific situations of concern—and then uploaded the footage to the web portal. After evaluation, clinicians diagnosed ASD when they uncovered sufficient examples of behaviours that met diagnostic criteria for the disorder [40,41,42]. On the other hand, many studies in the literature focused on the use of telemedicine in the treatment of ASD with encouraging results [35,43,44,45]. It is worth noting that the primary recipients of telemedicine intervention programmes are usually not ASD patients directly, but their parents, who then deliver them to their children [20]. Caregiver training can improve parent knowledge and lead to reductions in dysfunctional behaviours andimprovements in ASD children’s communication skills [46]. Interventions delivered remotely range from structured methods (e.g., interventions focused on principles of Applied Behaviour Analysis) [47] to more naturalistic approaches (e.g., Early Start Denver Model [48,49].

In the literature, many studies have examined the efficacy of remote approaches for ASD diagnosis and intervention [37,38,39,40,41,50,51], parental satisfaction [52,53,54], perceptions of telemedicine, and its potential benefits and limitations after using the service remotely [39,55,56,57,58,59]. Although some studies to date have examined healthcare professionals’ and parents’ views after experiencing remote services for ASD [39,53,54,55,58,59], few studies have explored the opinions about telemedicine that providers and users have before its use [56,60]. In particular, Salomone et al. [60] evaluated families’ propensity and refusal factors toward telemedicine services offered by their ASD clinic. Better computer skills and higher satisfaction with the clinic lead to greater acceptance of the remote service, while fear of not being able to communicate effectively with the physician and fear that the remote interaction may not be warm enough are associated with greater rejection. According to Iacono et al. [56], barriers to telemedicine among health professionals include their limited experience in the field, cyber barriers, and the prejudice that families prefer face-to-face services. It is worth noting that most of the articles on this topic refer to the pre-pandemic period. Since then, telemedicine has become a common practice, and we hypothesize that users’ perceptions may also have changed and that they are more willing to use it than before. With this survey, we therefore propose to explore this under-researched topic in more depth by distributing a questionnaire to both parents and healthcare professionals to make their viewpoints comparable. To our knowledge previously published works have examined attitudes toward telemedicine services for ASD separately, without making a comparison between the perceptions of the two groups. 

This study aims to compare the expectations and concerns that parents and healthcare professionals, who have never experienced remote service for ASD, have regarding the use of telemedicine to provide diagnoses and interventions. Comparing their opinions will allow us to better understand what expectations to focus on and what prejudices to overcome to improve the service so that a model can be created that addresses provider and user concerns.

## 2. Materials and Methods

### 2.1. Participants

A new questionnaire was specifically designed and manually delivered to participants to avoid excluding users who do not have appropriate devices or a good internet connection. The questionnaire was distributed from September to October 2021 to: 

(1) Healthcare professionals with expertise in diagnosing and caring of ASD patients working in the Child Neuropsychiatry Unit of the University Hospital “Policlinico di Bari”;

(2) Parents of children and adolescents (under 18 years) referred to the Child Neuropsychiatry Unit of the University Hospital” Policlinico of Bari” and diagnosed with ASD, in a traditional face-to-face method, in accordance with DSM-5 criteria. 

If both parents were present at the time of the request to complete the questionnaire, only one of them was asked tocomplete it.

A critical inclusion criteria was that the recruited participant (healthcare professional or parent) had never had any experience with the ASD telemedicine service before completing the questionnaire.

**Figure 1:**
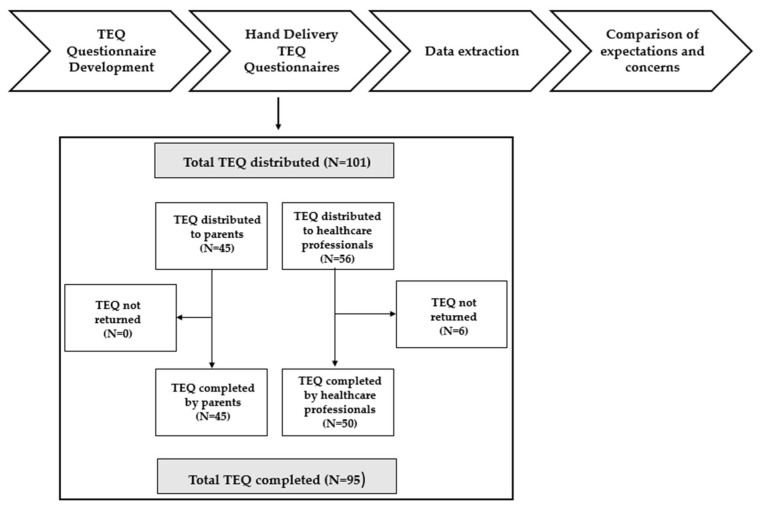
Workflow of the study. TEQ = Telemedicine Expectations Questionnaire.

Figure 1 illustrates the workflow of the present study.

### 2.2. Measures

#### 2.2.1. Socio-Demographic Information

Socio-demographic data of the participants were collected. Parent data included: gender, marital status, race, educational level, and family socioeconomic status (SES [61]). Parents were also asked to provide information about their children, such as age, gender, race, severity level of ASD, and ongoing therapies. Healthcare professionals provided socio-demographic information including gender, race, type of healthcare professional, and years of professional experience.

#### 2.2.2. Questionnaire

The survey questionnaire was specifically designed as a self-administered questionnaire and was based on previously published articles [39,56,59,60,62,63,64]. The Telemedicine Expectations Questionnaire (hereafter TEQ) consists of two sections: (1) expectations about the uses and potential benefits of telemedicine; and (2) concerns about objective and subjective barriers. 

The first section includes 16 items divided into two distinct subsections that assess (I) expectations of uses and willingness to telemedicine and (II) potential benefits of telemedicine using a 5-point Likert scale (from 1 = strongly agree to 5 = strongly disagree). The first subsection includes items on expectations for the use of telemedicine as a tool to provide diagnoses, interventions, and recommendations to families of patients with ASD, as well as potential integration with traditional face-to-face care. The last three items in this subsection examine the respondents’ willingness to use telemedicine in both routine and emergency situations. The second subsection includes items on the potential benefits of: improving parenting skills in managing children’s behaviour; reducing children’s behaviour problems; reducing the costs of accessing care; saving time; improving management of family routines; improving access to care; allowing both divorced parents to contribute to healthcare; and allowing the ASD child to be observed in the home setting. 

The second section consists of two closed questions that address objective barriers (lack of digital devices; lack of knowledge of digital technology; poor internet connection; active parental involvement/supervision during telemedicine services; presence of distractions at home; and that the camera is unable to follow the children as they move around), as well as subjective barriers to telemedicine use (severity of ASD; embarrassment of the child; changes in the child’s behaviour during telemedicine services; distraction of the ASD child by the use of digital devices; influence of the home environment on the child’s compliance; changes in the traditional doctor–patient relationship; potential negative impact of digital devices).

The questionnaire was accompanied by a note that clearly explained what the medical term “telemedicine” means, what uses exists in the diagnosis and treatment of ASD, and the aim of the survey. 

A clear and simplified vocabulary was intentionally used in the development of the questionnaire so that it could be understood by both healthcare professionals and parents, and care was taken to preserve the anonymity of respondents.

The wording of the questionnaire is both positive and negative to minimise bias. The internal consistency of the questionnaires was determined using Cronbach’s α and was 0.95, indicating strong consistency.

### 2.3. Statistical Analysis

To account for non-normality, data were evaluated through the Shapiro–Wilk test. Numerical variables related to expectations about telemedicine were reported as median and IQR and compared through Wilcoxon Rank Sum Test. Survey data regarding concerns about barriers were expressed as percentages and compared using the Chi-Square test. Statistical analysis was performed using R 4.0.2 (R Foundation for Statistical Computing, Vienna, Austria. Released on 22 June 2020). Statistical significance α was fixed to 0.05. 

## 3. Results

The questionnaire was distributed to 45 parents and 56 healthcare professionals. A total of 45 parents and 50 healthcare professionals returned the questionnaire, which corresponds to a response rate of 94.06%.

### 3.1. Respondents’ Description

The parents’ sample included 32 mothers and 13 fathers of children/adolescents with ASD (37 boys, 8 girls) aged between 2 and 18 years of age (m = 7.62 years, standard deviation (SD) = 4.36 years). Of these, 71% were diagnosed with ASD level 1. Of the 50 healthcare professionals who participated in the survey, 41 are child neuropsychiatrists, 6 are psychologists, 2 are speech therapists, and 1 is a professional educator. The characteristics of participants are presented in Table 1.

### 3.2. Expectations on the Uses and Potential Benefits of Telemedicine

As summarised in Table 2, our survey shows overall confidence of parents and healthcare professionals in the use of telemedicine and several common viewpoints can be identified. Both groups of respondents indicate a decided willingness to use telemedicine in emergency situations (*p* = 0.4588) and express positive views on its use for counselling and communication (*p* = 0.0598), to save costs (*p* = 0.2262) and time (*p* = 0.8223), and to allow the observation of the child in the home environment (*p* = 0.0726). Both parents and healthcare professionals believe that telemedicine is quite useful for the treatment of ASD (*p* = 0.2104), and they say they are quite willing to use it for diagnosis (*p* = 0.9509). Furthermore, both groups are also less confident about the effectiveness of telemedicine in reducing the behavioural problems of ASD children (*p* = 0.4219).

On the other hand, the comparison of the answers given by the respondents showed statistically significant differences. Parents have higher expectations of the use of telemedicine as an alternative (*p* = 0.0223) and a complement (*p* = 0.0061) to the traditional face-to-face diagnosis of ASD, as well as an adjunct to traditional intervention (*p* ≤ 0.0001). In terms of potential benefits, parents are more confident than healthcare professionals that telemedicine improves parenting skills in managing the children’s behavioural problems (*p* = 0.0147), improves management of family routines (*p* = 0.0034), increases flexibility in offering care (*p* = 0.0034), and allows divorced parents to participate in their child’s medical care (*p* = 0.0243). Although both parents and healthcare professionals state that they are willing to use telemedicine, our analysis shows that healthcare professionals are more willing than parents to use it as a routine tool for the care of children and adolescents with ASD (*p* = 0.0026). As can be seen in Table 3, mothers with a high level of education (*p* = 0.0432) and child neuropsychiatrists (*p* = 0.0193), especially those with more than 5 years of professional experience (*p* = 0.0023) expressed significantly more positive expectations about the use and willingness to use telemedicine. In addition, parents of children and adolescents with ASD level 1 answered significantly more positively to both the first (*p* = 0.0261) and second (*p* = 0.0327) subsections of the TEQ. The level of agreement, expressed as a percentage, between respondents for each item of the first section of the TEQ is presented in the Supplementary Material (Table S1).

### 3.3. Concerns about Objective and Subjective Barriers 

The survey shows that parents and healthcare professionals express some common concerns about the use of telemedicine services for ASD. Sources of major concern for both groups are the presence of distractions at home (*p* = 0.409), the distraction of ASD child due to the use of digital devices (*p* = 0.341), child’s compliance influenced by the home environment (*p* = 0.475), and changes in the traditional doctor–patient relationship (*p* = 0.488). Of less concern for both are the inability of the camera to adequately follow the child on the move (*p* = 0.308), the embarrassment of the child (*p* = 0.051), and the possible negative impact of digital devices (*p* = 0.064).

However, statistically significant differences were also found between the answers of the two groups of respondents (see Table 4). Compared to parents, healthcare professionals are significantly more concerned about objective barriers such as lack of digital devices (*p* = 0.000), lack of knowledge about digital technology (*p* = 0.000), poor internet connection (*p* = 0.006), and subjective barriers such as severity level of ASD (*p* = 0.000). Conversely, parents express significantly greater concerns than healthcare professionals about the need for active parental involvement/supervision during telemedicine services (*p* = 0.015) under the objective barriers, and about changes in ASD child’s behaviour during telemedicine services (*p* = 0.049) under the subjective barriers.

## 4. Discussion

In recent years, and particularly during the COVID-19 pandemic, a growing number of studies have investigated the attitudes of parents or healthcare professionals toward the use of telemedicine in the care of people with ASD [56,60,65,66,67]. However, little is known about the expectations and prejudices they have before having experienced remote service. This study aims to compare the expectations and concerns expressed by parents and health professionals about the use of telemedicine. The identification and correction of any critical issues, based on the users and providers’ opinions, are critical for improving telemedicine services [68]. 

### 4.1. Expectations on the Uses and Potential Benefits of Telemedicine

Although all respondents are undoubtedly aware of the remarkable potential of telemedicine, our study found some statistically significant differences between the responses of the two groups. On the one hand, parents of children and adolescents with ASD have significantly higher expectations of telemedicine both as a comprehensive diagnostic tool and as an integrated diagnostic tool; on the other hand, healthcare professionals express some concerns about its use, although they agree to use it, especially to provide ASD treatment. The slight distrust of healthcare professionals is in line with the results of the previous study by Dunlkey et al. [69], who found that healthcare professionals were more sceptical about a telematic delivery of health services than potential users. Interestingly, recent studies have shown that healthcare professionals, who had some concerns about telemedicine prior to its use, changed their minds about its potential [67] and put their fears in perspective [70] after they had experience with remote services. Since many studies were published before the pandemic, we hypothesise that the use of the technology, which was widely recorded in the health sector and beyond during the pandemic, could have changed providers and users’ opinions about it. Indeed, when we compare our results with those of Iacono et al. [56] in 2016, we find that telemedicine is viewed more optimistically as an adjunct to face-to-face rehabilitation care and there is less concern about potential changes in the doctor–patient relationship. However, among both parents and health professionals who participated in the present study, the lower scores indicating a of greater willingness to use telemedicine as a means of diagnosing or treating autism in emergencies or as an integration of face-to-face services rather than as a routine tool may suggest a greater reliance on traditional face-to-face care.

In our survey, healthcare professionals are also more aware than parents that exclusive use of telemedicine may not be helpful for making an accurate diagnosis of ASD. While telehealth is known to ensure the observation of parent–child interactions in their natural environment [71,72] and to protect ASD children from potentially stressful experiences (e.g., travel meltdowns, crowding, other stressful environmental elements in waiting rooms) [73,74], it does not allow for identification of behavioural problems that occur outside the home or with unfamiliar people. Indeed, children with ASD are often inflexible in terms of adherence to routine (sameness), and even small changes in their habits can lead to agitation [1]. Observing children exclusively in a predictable and home-like environment could mask these dysfunctional behaviours. According to the literature, most healthcare professionals believe that telemedicine cannot replace face-to-face interaction and that changes to traditional practises are needed to adapt them for telemedicine [75]. Although the American Telemedicine Association has issued general guidelines for the use of telemedicine in mental health assessment [76], similar specific guidelines have not yet been developed for the ASD population, nor have standardised tools been specifically developed and validated specifically for the diagnosis of ASD using only technological modalities. For example, although ADOS is considered one of the “gold standards” for diagnosing ASD in face-to-face settings, not all of its items are appropriate for remote use (e.g., the imitation task, in which the ASD child is asked to repeat actions and behaviours performed just before by the healthcare professional) [75]. However, a new telematic assessment tool for ASD, called the Brief Observation of Symptoms of Autism (BOSA), has been developed and adapted by ADOS [77]. The mistrust of healthcare professionals could also be motivated by the awareness that the use of telemedicine would lead to additional difficulties and efforts in clinical practice, as they need to be trained and constantly updated on these new methods of service delivery. In addition, they also need to to prepare and send instructions and educational materials to parents prior to the virtual appointment. Parents, through parent training programs delivered by telematics systems, mediate intervention on their children [20,73,74,75,78,79,80], thus ensuring a more family-centred approach to care [81,82]. In contrast, few studies focus on distance interventions that directly target children and adolescents with ASD. As a result, little is known about the perceptions of telemedicine and the response to these services by peolpe with ASD [44,83,84,85]. What is certain is that some children and adolescents with ASD spend a great deal of time using computing devices for recreational purposes [86,87]. Because they find the technology familiar and comfortable, they could consider telematic interventions as engaging, and preferable to in-person treatment [88]. Another finding of our survey is that parents have greater expectations than healthcare professionals about the potential of telemedicine to improve parenting skills in managing their children’s problem behaviours. Indeed, previous studies have found that parents acquire knowledge to reduce their children’s maladaptive behaviours after a parent-mediated intervention based on telemedicine [63,89,90,91,92,93]. In contrast, both parents and healthcare professionals have low expectations for the effectiveness of telemedicine in reducing problem behaviours in children and adolescents with ASD, although several studies have confirmed improvements in this area as well [93,94,95]. For example, Wacker and others [93] found that the average reduction in problem behaviours was 93.5%. Likewise, Lindgren et al. [94] found that problem behaviours in their sample decreased by an average of 90% after telematic treatments. Parents’ higher expectations of telemedicine’s potential to improve parenting skills compared with those to improve children’s problem behaviours reflect the difficulties and frustration daily experienced by parents of children with ASD. Even thoughsome maladaptive behaviours do not yield much benefit despite therapy, parents hope telemedicine will provide the right suggestions to better to contain and manage them. In our survey, families also believe that telemedicine offers greater organizational benefits than those recognized by health professionals, as it improves the management of family routines, and favours the participation of families, including divorced ones, in appointments with healthcare professionals. It is worth noting that caring for an ASD child can be physically and emotionally exhausting, as parents find it difficult to cope with their ASD children, as they feel the pressure of having to meet everyone’s needs, and are often forced to give up their jobs as well in order to devote their full attention to the child. Consequently, parents of children and adolescents with ASD experience higher levels of stress than those dealing with offspring without disabilities [96] or with other disabilities [97]. Specifically, most research has found that mothers are more stressed than fathers in raising their offspring with ASD [98,99,100], although this finding is likely influenced by the mother’s greater involvement in family management and the greater amount of time they spend with their children with ASD [101]. In addition, the high expectations of respondents from both groups studied regarding time and cost saving that families could achieve through telemedicine are consistent with the findings of previous studies [38,56,94]. Transportation costs and work loss due to paternal leave may have a telling impact on the family’s economic situation. The use of telemedicine would significantly reduce the burden of parents as they would have to spend less time travelling to the clinic. Although there is no doubt that telemedicine reduces healthcare costs globally, it is important to pointing out that there are still issues that persist about reimbursement for remote healthcare services in countries still lacking universal healthcare covarage. In fact, while in Italy and in other European countries, telemedicine is included in the essential levels of assistance provided by the National Health Service and all citizens benefit from free or low-cost health services, in countries with private health insurance systems, the use of telemedicine is limited by restrictions imposed by insurance companies, which do not pay for all remote services [102].

### 4.2. Concerns about Objective and Subjective Barriers

Regarding the perceived barriers, our survey shows that health professionals are more concerned than parents about potential objective obstacles to the delivery of telemedicine services, such as lack of digital devices, lack of knowledge of digital technology, and poor-quality Internet connection. These technological issues are widely viewed in the literature as barriers to telemedicine adoption [20,31,39,56,70,103,104]. It is recommended and necessary to check if the connection and device are available and adequate, before starting telematics services [105,106]. Although the supply of broadband services has increased significantly in recent decades, there are still areas that do not have enough Internet coverage, leading to the digital divide [107,108]. A slow Internet connection compromises the quality of the video and undermines the effectiveness of the remote service as observation of facial expressions and behaviour of children with ASD plays a critical role in clinical assessment. Furthermore, some families do not have the financial resources to afford an unlimited Internet use or Wi-Fi Internet connections. In addition, health systems are probably not yet well equipped to deliver health services remotely, and telemedicine health activities are still inconsistent across the countries. This likely contributes to scepticism of health professionals. Future initiatives should focus on developing answers to these challenges, also creating dedicated telemedicine points of service, from which families can more easily connect to the hospital [94,109]. The present study also found that parents were more concerned than healthcare professionals about not having enough time to devote to remote services. Although active participation in assessment procedures is valued by most parents [59], it requires demanding efforts for families with ASD children (e.g., parental presence during the virtual visit, study of materials previously sent by the health professionals, preparation of an adequate setting within the home environment). Among the subjective barriers, compared to parents, healthcare professionals consider that the level of severity of autism is a limiting factor for the use of telemedicine. Therefore, they are more aware of the importance of selecting children/adolescents with ASD as recipients of a remote service. A physical disability (e.g., deafness, blindness, infantile cerebral palsy, etc.) may prevent the ability to participate in virtual interventions. Due to hypersensitiviy or hyposensitivity to sensory inputs, visual and auditory stimuli (e.g., excessive brightness, flashing screens, excessive loud sounds, etc.) can distract or disturb some individuals with ASD. Notably, comorbidities such as photosensitive epilepsy limit the use of flashing screens [110]. The use of telemedicine should be tailored according to the characteristics of the service users. If the child/adolescent with ASD has good cognitive, communication, and technological skills, as well as an appropriate level of comfort and cooperation, they may not need the caregiver mediation [85]. An individual with ASD with a higher level of severity may not be sufficiently cooperative and indirect remote assistance may be required. No less important is the assessment of the family environment before deciding whether the ASD assessment should be provided remotely or not, to adopt an individualised approach according to the needs and resources of the family. Regarding subjective barriers, parents are significantly more concerned than healthcare professionals about the possible change in their children’s behaviour during telematic sessions. Although, on the one hand, awareness of being observed may lead to reactivity in ASD children [111], on the other hand, provision of health services at a distance could alleviate children’s anxiety and stress allowing them to remain in a predictable and familiar environment [56,88]. Consistent with the available literature, approximately half of the parents and professionals in our study sample expressed concern about the child’s distraction induced by electronic devices and the home environment (e.g., sharing rooms with siblings, presence of young children or other family members, etc.) [39,56,57,58], which may reduce their willingness to participate in the diagnostic/therapeutic session. In light of these concerns, healthcare professionals should provide practical advice to families on the proper use of the devices and the optimal preparation of the home setting (limiting distractions at home, materials to be used during telematics sessions). Another issue highlighted by our interviewees is the change in the traditional doctor–patient relationship. In telemedicine, the physical distance between interlocutors and the interposition of a screen do not guarantee adequate synchronisation of gestures and words, which is essential for proper interaction and mutual understanding [112], and less intimacy in the doctor–patient relationship could be perceived. However, in a study by Doyen et al. on telematic assessment of children with ASD, parents perceived greater responsibility and ability to make decisions independently, which strengthened the doctor–patient relationship [113]. It should be noted, however, that telemedicine today is not an alternative, but rather a complement to traditional face-to-face services. People with ASD need a healthcare professional who encourages and continually draws attention to them. A telematics-only approach should not replace face-to-face interventions that also aim to improve the socialisation and integration skills of peolpe with ASD.Finally, families often must wait months to years for their child to receive an ASD diagnosis and specific treatment [2,114] even though it is now known that early intervention plays a key role in maximising the learning for children with ASD when the brain is still developing. Universal screening, while desirable to reduce the age of ASD diagnosis, is nevertheless difficult to achieve [115]. Similarly, geographic and cultural barriers as well as the scarcity of resources and specialised health professionals, contribute to this delay [32,116]. For all these reasons, a telemedicine service for ASD may also contribute to faster diagnostic assessment [109].

### 4.3. Limits

One of the major limitations of this study is the limited number of parents and healthcare professionals that may have skewed the results and overestated our conclusions. Moreover, single-hospital recruitment and the greater representativeness of level 1 severity of ASD among children as well as child neuropsychiatrists among healthcare professionals may have led to a bias in the selection of the study sample. 

## 5. Conclusions

Overall, our survey shows that families with children/adolescents with ASD and healthcare professionals have different views of telemedicine as a health modality. Parents exhibited a greater propensity to employ telemedicine for the diagnosis and treatment of ASD, even as an integration for the traditional face-to-face method, and they also recognized potential benefits to be derived from its implementation. Conversely, healthcare professionals expressed greater concerns primarily related to potential objective barriers that might limit the utilization of telehealth services.

Our findings can serve as a starting point for identifying other variables that could motivate families and healthcare professionals to use telemedicine services for ASD, as well as barriers that might be perceived and, if possible, should be addressed. To develop an optimal and promising model of health service for ASD, it is necessary that the telemedicine service tries to meet the preferences of health professionals and families, taking into account the resources and needs of both of them. There is little doubt that telemedicine is not suited for all patients with ASD and that it cannot be used in all scenarios. However, there is sufficient evidence that telemedicine provides several advantages. For example, it could be used to provide adequate training for families, which has been shown to be one of the most relevant needs of parents of children with ASD. Giving parents practical and concrete counsel on how to manage everyday life, also using digital devices that we have become accustomed to, especially during the pandemic, could considerably improve their quality of life and reduce the burden on caregivers in these families. Undoubtedly, the use of telemedicine takes great efforts from professionals and family members. Modeling health interventions based on their needs and expectations would allow us to lay the foundation for a new doctor–caregiver alliance that would be a real strength in the complex process of caring for a child with ASD.

Since the aim of this study was to assess the expectations and preconceptions that parents and health professionals have before they have experience using telemedicine services for ASD, it would be interesting to reassess the opinions of the study sample after a period of experience with telemedicine to know how their views might change. Future studies should be conducted on larger samples to gather more reliable data, including a more representative group of families, ASD patients, and healthcare professionals. Considering that to promote health projects for ASD that are valid, effective, and valued, the expectations and concerns of healthcare professionals, parents, and ASD patients must be taken into account, future research should consider the expectations, concerns, desires, and needs of children and adolescents with ASD who are the primary stakeholders in the healthcare service.

## Figures and Tables

**Table 1:** Respondents’ characteristics. ASD—autism spectrum disorder.

**Healthcare Professionals (n = 50)**	
** Male, n (%)**	3 (6%)
** Race, n (%)**	
White	50 (100%)
Black	0 (0%)
Others	0 (0%)
** Type of healthcare professionals**	
Child neuropsychiatrist	41 (82%)
Psychologists	6 (12%)
Speech therapists	2 (4%)
Professional educators	1 (2%)
** Years of professional experience, n (%)**	
1–5	27 (54%)
6–10	12 (24%)
11–20	7 (14%)
>20	4 (8%)
**Parents (n = 45)**	
** Male, n (%)**	13 (29%)
** Marital status, n (%)**	
Married	42 (94%)
Single	0 (0%)
Divorced	1 (2%)
Separeted	2 (4%)
** Race, n (%)**	
White	45 (100%)
Black	0 (0%)
Others	0 (0%)
**ASD Children (n = 45)**	
** Age, years; n (%)**	
<5	16 (36%)
6–10	20 (44%)
11–15	5 (11%)
16–18	4 (9%)
** Male, n (%)**	37 (82%)
** Race, n (%)**	
White	45 (100%)
Black	0 (0%)
Others	0 (0%)
** Severity level of ASD, n (%)**	
1	32(71%)
2	7 (16%)
3	6 (13%)
** Pharmacological interventions, n (%)**	
None	35 (78%)
Antipsychotics	7 (15%)
Antidepressants	1 (2%)
Antiepileptics	3 (7%)
** Non-pharmacological interventions, n (%)**	
None	11 (24%)
Psychomotor therapy	9 (20%)
Speech therapy	11 (25%)
Cognitive–behavioural therapy	17 (38%)
Psychotherapy	4 (9%)

**Table 2:** Results of Wilcoxon Rank Sum Test for the comparison of parents’ and professionals’ answers on the first section of Telemedicine Expectations Questionnaire: expectations on the uses and potential benefits of telemedicine.

	Statement	Parents (n = 45)		HP (n = 50)		
		Mean (SD)	Median (IQR)	Mean (SD)	Median (IQR)	*p*-Value
**USES AND WILLINGNESS**	Telemedicine is a useful tool for diagnosing ASD	2.98 (0.94)	3 (2–4) *	3.4 (0.76)	3 (3–4)	**0.0223**
	b. Telemedicine is a useful tool for treating ASD	3.09 (0.97)	3 (2–4)	3.32 (0.87)	3 (3–4)	0.2104
	c. Telemedicine is a useful tool for communicating diagnoses or providing recommendations to families	1.93 (0.96)	2 (1–3)	2.26 (0.90)	2 (2–3)	0.0598
	d. Telemedicine is a useful integration into traditional face-to-face diagnosis	2.11 (1.21)	2 (1–3) *	2.62 (0.83)	3 (2–3)	**0.0061**
	e. Telemedicine is a useful integration into traditional face-to-face treatment	2.13 (1.10)	2 (1–3) *	2.84 (0.93)	3 (2–4)	**<0.0001**
	f. I am willing to use telemedicine as a routine tool for the diagnosis of ASD	2.98 (1.18)	3 (2–4)	2.98 (1.33)	3 (2–4)	0.9509
	g. I am willing to use telemedicine as a routine tool for the treatment ofASD	3.09 (1.08)	3 (2–4)	2.36 (1.17)	2 (1–3) *	**0.0026**
	h. I am willing to use telemedicine for the diagnosis and treatment of ASD only in emergency situations	1.69 (1.18)	1 (1–2)	1.82 (1.22)	1 (1–2)	0.4588
	**Total a–h**	20 (7.41)	19 (14–25)	21.6 (6.21)	22 (17–26)	0.1445
**POTENTIAL BENEFITS**	i. Telemedicine is a useful tool for improving parenting skills in managing behavioural problems in children and adolescents with ASD	2.31 (1.26)	2 (1–3) *	2.82 (0.80)	3 (2–3)	**0.0147**
	j. Telemedicine is a useful tool for reducing behavioural problems of children and adolescents with ASD at home	3.22 (1.22)	3 (3–4)	3.52 (0.76)	4 (3–4)	0.4219
	k. Telemedicine reduces costs for accessing care (e.g., travel time, transportation expenses, missed work)	2.02 (1.25)	2 (1–3)	2.06 (0.79)	2 (2–2)	0.2262
	l. Telemedicine saves time (waiting time in clinics, travel time to the centre, etc.)	1.91 (1.18)	1 (1–2)	1.78 (0.76)	2 (1–2)	0.8223
	m. Telemedicine improves the management of family routine	2.04 (1.22)	2 (1–3) *	2.62 (0.88)	3 (2–3)	**0.0034**
	n. Telemedicine increases flexibility in offering care services	1.93 (1.19)	2 (1–2) *	2.42 (0.90)	2 (2–3)	**0.0034**
	o. Telemedicine allows both divorced parents to contribute to their child’s assessment or intervention	1.89 (1.13)	2 (1–2) *	2.16 (0.77)	2 (2–3)	**0.0243**
	p. Telemedicine allows the ASD child to be observed in the home environment	1.89 (1.04)	2 (1–3)	2.16 (0.82)	2 (2–3)	0.0726
	**Total i–p**	17.22 (8.23)	16 (11–23) *	19.54 (4.63)	20 (17–22)	**0.0207**

ASD—autism spectrum disorder; HP—healthcare professionals; sd—standard deviation; in bold < *p*-value < 0.05; * significantly lower scores.

**Table 3:** Comparison of respondents’ answers using Wilcoxon Rank Sum Test.

**Parents’s Data (n = 45)**	**Total a–h**				**Total a–e**			**Total i–p**	
	**Mean (sd)**	**Median (IQR)**	***p***	**Mean (sd)**	**Median (IQR)**	***p***	**Mean (sd)**	**Median (IQR)**	***p***
**Respondent**			0.4066			0.3712			0.1913
Mother	19.44 (7.28)	18.0 (9.5)		11.91 (4.68)	11.0 (6.3)		16.44 (8.32)	14.5 (9.5)	
Father	20.33 (6.55)	20.0 (11.0)		12.50 (4.13)	12.0 (8.0)		18.67 (7.40)	19.0 (14.0)	
**Mother’s education level**			**0.0432**			0.0607			0.0531
Primary school	0	0		0	0		0	0	
Middle school	24.38 (7.70)	23.5 (10.0)		15 (4.92)	14.0 (6.0)		23.00 (8.90)	23.0 (12.8)	
High school	19.76 (7.34)	18.0 (11.0)		12.05 (4.56)	11.0 (8.0)		16.95 (8.36)	16.0 (10.0)	
Bachelor’s degree	28.50 (5.50)	28.5 (5.5)		17.00 (3.00)	17.0 (3.0)		23.00 (2.00)	23.0 (2.0)	
Master’s degree/postgraduate course/PhD	16.64 (4.59)	16.0 (7.8)		10.29 (3.39)	10.0 (5.0)		13.50 (4.94)	11.0 (5.8)	
**Father’s education level**			0.1079			0.1347			0.0838
Primary school	24.00 (5.00)	24.0 (5.0)		15.00 (2.00)	15.0 (2.0)		20.00 (5.00)	20.0 (5.0)	
Middle school	23.11(7.49)	20.0 (10.0)		14.11 (4.75)	12.0 (6.0)		20.44(9.50)	17.0 (14.0)	
High school	21.00 (7.60)	21.0 (12.0)		12.88 (4.92)	12.0 (8.0)		19.06 (8.18)	19.0 (14.0)	
Bachelor’s degree	0	0		0	0		0	0	
Master’s degree/postgraduate course/PhD	16.88 (5.88)	16.0 (8.0)		10.29 (3.67)	9.0 (5.0)		13.35 (5.73)	11.0 (7.0)	
**Family SES**			0.4510			0.4814			0.5982
Low	21.40 (5.54)	19.0 (11.0)		13.20 (3.37)	13.0 (7.0)		17.80 (4.83)	17.0 (6.0)	
Medium–low	20.22 (5.75)	20.0 (9.0)		12.33 (3.86)	12.0 (4.0)		18.11 (7.89)	18.0 (17.0)	
Medium	21.70 (7.75)	21.0 (12.25)		13.00 (4.45)	13.0 (7.0)		18.90 (7.67)	18.5 (13.3)	
Medium– high	20.46 (9.01)	17.0 (7.0)		12.77 (5.77)	12.0 (6.0)		17.46 (10.20)	13.0 (10.0)	
High	16.00 (4.21)	14.5 (7.3)		9.75 (3.27)	8.5 (4.3)		13.38 (5.07)	11.0 (6.5)	
**ASD children’s data (n = 45)**		**Total a–h**			**Total a–e**			**Total i–p**	
		**Median (IQR)**	***p***		**Median (IQR)**	***p***		**Median (IQR)**	***p***
**Age**			0.0711			0.0688			0.2219
≤6 year	18.38 (6.88)	16.0 (7.8)		11.04 (4.15)	10.5 (5.0)		15.63 (7.04)	12.0 (9.3)	
>6 year	21.86 (7.39)	21.0 (10.0)		13.62 (4.80)	13.0 (6.0)		19.05 (8.89)	17.0 (10.0)	
**Gender**			0.4295			0.3783			0.3329
Female	21.75 (7.61)	20.0 (3.0)		13.75 (5.02)	12.5 (3.8)		19.88 (8.65)	18.5 (5.0)	
Male	19.62 (7.21)	17.0 (12.0)		11.92 (4.49)	11.0 (8.0)		16.65 (7.91)	14.0 (12.0)	
**Pharmacological therapy**			0.3656			0.3721			0.1623
No	19.81 (7.31)	18.0 (10.0)		11.97 (4.54)	11.0 (7.0)		16.59 (7.77)	15.0 (10.0)	
Yes	21.90 (7.80)	20.0 (10.0)		13.60 (5.28)	12.5 (7.0)		20.70 (9.08)	18.5 (11.8)	
**Severity level of ASD**			**0.0261**			**0.0377**			**0.0327**
1	18.1 (5.1)	17.5 (7.5)		11.21 (3.40)	11.0 (5.5)		15.36 (6.05)	13.0 (8.3)	
2–3	24.4 (8.3)	26.0 (12.0)		14.69 (5.25)	15.0 (10.0)		21.54 (8.67)	21.0 (10.0)	
**Healthcare professionals’ data**		**Total a–h**			**Total a–e**			**Total i–p**	
		**Median (IQR)**	***p***		**Median (IQR)**	***p***		**Median (IQR)**	***p***
**Child neuropsychiatrist**			**0.0193**			**0.0079**			0.0510
Not	26.11 (5.63)	26.0 (3.0)		16.89 (2.88)	17.0 (3.0)		21.44 (4.78)	22.0 (3.0)	
Yes	20.61 (5.81)	19.0 (9.0)		13.90 (3.06)	14.0 (4.0)		19.12 (4.49)	19.0 (5.0)	
**Gender**			0.5529			0.5087			0.2958
Female	21.74 (6.19)	22.0 (8.5)		22.21 (8.7)	16.0 (5.0)		19.40 (4.66)	19.0 (5.0)	
Male	19.33 (4.99)	18.0 (6.0)		18.00 (3.74)	14.0 (3.0)		21.67 (2.05)	22.0 (2.5)	
**More than 5 years of professional experience**			**0.0023**			**0.0018**			0.0663
Not	19.04 (4.99)	18.0 (6.5)		16.26 (3.35)	13.0 (4.0)		18.33 (3.90)	19.0 (6.0)	
Yes	24.61 (6.01)	25.0 (7.0)		28.65 (7.93)	16.0 (4.0)		20.96 (4.90)	21.0 (6.0)	

ASD—autism spectrum disorder; SES—socioeconomic status; sd—standard deviation; in bold—*p*-value < 0.05.

**Table 4:** Comparison of parents’ and professionals’ answers on the second section of Telemedicine Expectations Questionnaire: Concerns about objective and subjective barriers.

Barriers	Statement	Parents	Healthcare Professionals	Pearson’s Chi-Square Test	*p*
**Objective**	Lack of digital devices	9%	70%	36.551	**0.000**
	Lack of knowledge of digital technology	25%	84%	34.059	**0.000**
	Poor-quality internet connection	36%	64%	7.666	**0.006**
	Active involvement/supervision of parents during telemedicine services	20%	4%	5.922	**0.015**
	Presence of at-home distractions	42%	34%	0.680	0.409
	Camera is unable to follow children as they moved around	24%	34%	1.040	0.308
**Subjective**	Severity level of ASD	22%	30%	12.418	**0.000**
	ASD child’s embarrassment during telemedicine services	22%	8%	3.813	0.051
	Changes in ASD child’s behaviour during telemedicine services	33%	16%	3.878	**0.049**
	Distraction of ASD child due to the use of digital devices	58%	48%	0.908	0.341
	ASD child’s compliance influenced by the home setting	53%	46%	0.510	0.475
	Changes in the traditional doctor-patient relationship	49%	54%	0.480	0.488
	Potential negative effects of digital devices	9%	26%	3.419	0.064
	No barrier	6%	2%	1.279	0.258

ASD—autism spectrum disorder; in bold—*p*-value < 0.05.

## Data Availability

The data used to support the findings of this study are included within the Supplemental Information file.

## Supplementary Materials

The following supporting information can be downloaded at: https://www.mdpi.com/article/10.3390/jcm11123294/s1. Table S1. Level of agreement, expressed in percentages, between the respondents for each item of the first section of TEQ.
