# Supplementary material for: Expectations and Concerns about the Use of Telemedicine for Autism Spectrum Disorder: A Cross-Sectional Survey of Parents and Healthcare Professionals
Source: J Clin Med. 2022 Jun 8;11(12):3294. doi: 10.3390/jcm11123294 (PMC9224762; doi:10.3390/jcm11123294)
Supplement: Supplementary file 1 [file jcm-11-03294-s001.zip › jcm-1708677-supplementary.pdf]

SUPPLEMENTAL MATERIAL

		Statement	respondent	LEVEL OF AGREEMENT				
				1 strongly agree	2 agree	3 neither agree nor disagree	4 disagree	5 strongly disagree
USES AND WILLINGNESS	a.	Telemedicine is a useful tool for diagnosing ASD	parents	4%	27%	40%	25%	4%
			healthcare professionals	0%	10%	46%	38%	6%
	b.	Telemedicine is a useful tool for treating ASD	parents	4%	22%	40%	27%	7%
			healthcare professionals	2%	14%	40%	38%	6%
	c.	Telemedicine is a useful tool for communicating diagnoses or providing recommendations to families	parents	40%	33%	23%	2%	2%
			healthcare professionals	6%	40%	42%	10%	2%
	d.	Telemedicine is a useful integration into traditional face-to-face diagnosis	parents	42%	25%	18%	11%	4%
			healthcare professionals	6%	32%	36%	24%	2%
	e.	Telemedicine is a useful integration into traditional face-to-face treatment	parents	36%	31%	20%	11%	2%
			healthcare professionals	20%	42%	32%	4%	2%
	f.	I am willing to use telemedicine as a routine tool for the diagnosis of ASD	parents	9%	31%	25%	24%	11%
			healthcare professionals	10%	24%	34%	22%	10%
	g.	I am willing to use telemedicine as a routine tool for the treatment of ASD	parents	2%	36%	24%	27%	11%
			healthcare professionals	28%	32%	20%	16%	4%
	h.	I am willing to use telemedicine for the diagnosis and treatment of ASD only in emergency situations	parents	69%	9%	11%	7%	4%
			healthcare professionals	60%	16%	12%	6%	6%
	i.	Telemedicine is a useful tool for improving parenting skills in managing behavioural problems in children and adolescents with ASD	parents	36%	24%	18%	18%	4%
			healthcare professionals	4%	30%	46%	20%	0%
	j.	Telemedicine is a useful tool for reducing behavioural problems of children and adolescents with ASD at home	parents	15%	7%	29%	38%	11%
			healthcare professionals	0%	8%	40%	44%	8%

**POTENTIAL BENEFITS**	k.	Telemedicine reduces costs for accessing care (e.g., travel time, transportation expenses, missed work)	**parents**	47%	27%	11%	9%	6%
			**healthcare Professionals**	22%	54%	22%	0%	2%
	l.	Telemedicine saves time (waiting time in clinics, travel time to the centre, etc.)	**parents**	51%	25%	11%	9%	4%
			**healthcare professionals**	40%	44%	14%	2%	0%
	m.	Telemedicine improves the management of family routine	**parents**	47%	22%	16%	11%	4%
			**healthcare professionals**	12%	28%	46%	14%	0%
	n.	Telemedicine increases flexibility in offering care services	**parents**	49%	27%	13%	4%	7%
			**healthcare professionals**	14%	42%	34%	8%	2%
	o.	Telemedicine allows both divorced parents to contribute to their child's assessment or intervention	**parents**	49%	29%	11%	7%	4%
			**healthcare professionals**	18%	52%	26%	4%	0%
	p.	Telemedicine allows the ASD child to be observed in the home environment	**parents**	49%	22%	22%	5%	2%
			**healthcare professionals**	24%	38%	36%	2%	0%

Table S1. Level of agreement, expressed in percentages, between the respondents for each item of the first section of TEQ
